# Comprehensive Metabolic Profiling of Inflammation Indicated Key Roles of Glycerophospholipid and Arginine Metabolism in Coronary Artery Disease

**DOI:** 10.3389/fimmu.2022.829425

**Published:** 2022-03-08

**Authors:** Qian Zhu, Yonglin Wu, Jinxia Mai, Gongjie Guo, Jinxiu Meng, Xianhong Fang, Xiaoping Chen, Chen Liu, Shilong Zhong

**Affiliations:** ^1^School of Medicine, South China University of Technology, Guangzhou, China; ^2^Department of Pharmacy, Guangdong Provincial People’s Hospital, Guangdong Academy of Medical Sciences, Guangzhou, China; ^3^School of Pharmaceutical Sciences, Southern Medical University, Guangzhou, China; ^4^Guangdong Provincial Key Laboratory of Coronary Heart Disease Prevention, Guangdong Cardiovascular Institute, Guangdong Provincial People’s Hospital, Guangdong Academy of Medical Sciences, Guangzhou, China; ^5^Department of Cardiology, Guangdong Cardiovascular Institute, Guangdong Provincial People’s Hospital, Guangdong Academy of Medical Sciences, Guangzhou, China; ^6^Department of Clinical Pharmacology, Xiangya Hospital, Central South University, Changsha, China; ^7^Department of Cardiology, The First Affiliated Hospital of Sun Yat-sen University, Guangzhou, China

**Keywords:** coronary artery disease, metabolome, lipidome, inflammation, glycerophospholipid metabolism, arteriosclerosis

## Abstract

**Background:**

Systemic immune inflammation is a key mediator in the progression of coronary artery disease (CAD), concerning various metabolic and lipid changes. In this study, the relationship between the inflammatory index and metabolic profile in patients with CAD was investigated to provide deep insights into metabolic disturbances related to inflammation.

**Methods:**

Widely targeted plasma metabolomic and lipidomic profiling was performed in 1,234 patients with CAD. Laboratory circulating inflammatory markers were mainly used to define general systemic immune and low-grade inflammatory states. Multivariable-adjusted linear regression was adopted to assess the associations between 860 metabolites and 7 inflammatory markers. Least absolute shrinkage and selection operator (LASSO) logistic-based classifiers and multivariable logistic regression were applied to identify biomarkers of inflammatory states and develop models for discriminating an advanced inflammatory state.

**Results:**

Multiple metabolites and lipid species were linearly associated with the seven inflammatory markers [false discovery rate (FDR) <0.05]. LASSO and multivariable-adjusted logistic regression analysis identified significant associations between 45 metabolites and systemic immune-inflammation index, 46 metabolites and neutrophil–lymphocyte ratio states, 32 metabolites and low-grade inflammation score, and 26 metabolites and high-sensitivity C-reactive protein states (*P* < 0.05). Glycerophospholipid metabolism and arginine and proline metabolism were determined as key altered metabolic pathways for systemic immune and low-grade inflammatory states. Predictive models based solely on metabolite combinations showed feasibility (area under the curve: 0.81 to 0.88) for discriminating the four parameters that represent inflammatory states and were successfully validated using a validation cohort. The inflammation-associated metabolite, namely, β-pseudouridine, was related to carotid and coronary arteriosclerosis indicators (*P* < 0.05).

**Conclusions:**

This study provides further information on the relationship between plasma metabolite profiles and inflammatory states represented by various inflammatory markers in CAD. These metabolic markers provide potential insights into pathological changes during CAD progression and may aid in the development of therapeutic targets.

## Introduction

Coronary artery disease (CAD) is one of the leading causes of morbidity and mortality worldwide (1), and its common underlying pathology is atherosclerosis. Systemic inflammation affecting the media and intima layers of arteries is a predominant characteristic that contributes to atherosclerosis and is maladaptively triggered by metabolic alterations and lipid accumulation in the arterial wall (2, 3). Although the ability in risk factor recognition and medical treatment have substantially improved, atherosclerotic CAD remains a serious clinical problem, and its underlying molecular mechanisms involving the inflammatory process are poorly understood. Therefore, the relationship between inflammation and metabolic alterations in CAD must be investigated.

Numerous circulating inflammatory markers have been proposed, including high-sensitivity C-reactive protein (hs-CRP), white blood cell count (WBC), and fibrinogen (FIB), which are important for increased risks in patients with CAD (4, 5). Their elevated levels indicate chronic low-grade inflammation, which, in turn, is associated with many metabolic disturbances (6) and aggravates atherogenesis (7). CRP and FIB are acute-phase reactants induced by inflammatory cytokines and exert proinflammatory effects on the vascular endothelium to promote thrombotic states (8, 9). Another accessible inflammatory marker, WBC, is an independent predictor of hospitalization for heart failure, acute myocardial infarction, and all-cause death in patients with CAD (10). A low-grade inflammation (INFLA) score, which comprises hs-CRP, WBC, platelet count, and granulocyte-to-lymphocyte ratio (11), has been used in the Moli-sani cohort and is independently associated with adverse health risks, particularly in individuals with diabetes and cardiovascular disease (CVD) (12). Although low-grade inflammatory markers have been extensively studied, their relationship with atherosclerosis remains controversial (5). Novel CVD prognostic inflammatory markers based on immune circulatory cells have been proposed, including systemic immune-inflammation index (SII), neutrophil–lymphocyte ratio (NLR), and platelet–lymphocyte ratio (PLR). NLR and PLR are strongly associated with CAD severity and clinical outcome (13–15), and SII integrates peripheral platelet, neutrophil, and lymphocyte counts to better reflect the balance between the host inflammation and immune situation (16). SII is associated with coronary severity (17) and provides a better prediction of major cardiovascular events and mortality than conventional risk factors do in patients with CAD (18). Thus, low-grade inflammatory markers and inflammatory indices based on immune cells play a pivotal role in CAD development.

The immune cells involved in inflammation undergo obvious metabolic changes that influence their functions and disease development (19). Owing to its close link to metabolic perturbation, the metabolic implications of inflammation must be urgently analyzed. Metabolomics is a suitable tool for the systematic investigation of functional molecules in body fluids. Previous metabolomic studies have identified the metabolic changes associated with the urea cycle and oxidative stress associated with low-grade inflammatory markers in healthy individuals (20) and patients with rheumatoid arthritis (21). However, the relationship of plasma metabolic profiles to low-grade and immune-cell-based inflammatory markers in CAD and their contribution to atherosclerosis remains poorly examined.

This study aimed to explore the relationship of metabolomics and lipidomics to seven inflammatory markers in patients with CAD to obtain further insights into inflammation-associated metabolism during disease progression. The results showed that multiple metabolic signatures are associated with low-grade and immune cell-based inflammatory markers in CAD. Glycerophospholipid metabolism and arginine and proline metabolism, respectively, are the key altered metabolic pathways. Moreover, inflammation-associated metabolites are correlated with atherosclerosis.

## Materials and Methods

### Study Population

**Figure 1:**
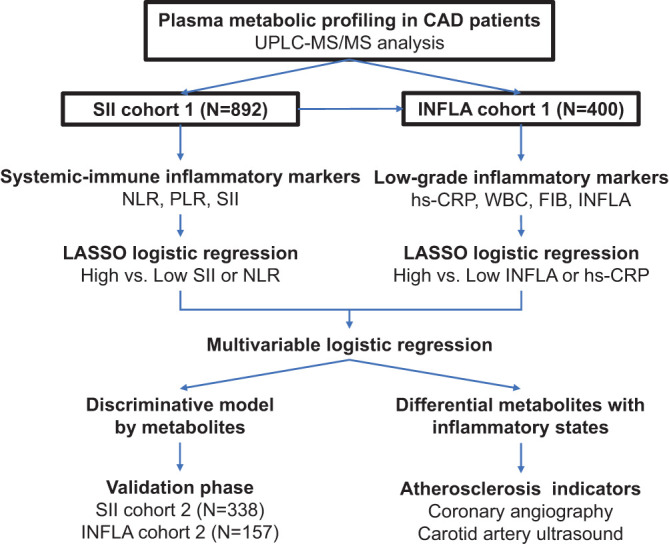
Overview of the flow chart for study design and data analysis. CAD, coronary artery disease; SII, systemic immune-inflammation index; NLR, neutrophil–lymphocyte ratio; PLR, platelet–lymphocyte ratio; hs-CRP, high-sensitivity C-reactive protein; WBC, white blood cell count; FIB, fibrinogen; INFLA score, low-grade inflammation score; LASSO, least absolute shrinkage and selection operator; UPLC–MS/MS, ultra-performance liquid chromatography–tandem mass spectrometry.

The study flow chart is shown in **Figure 1**. This two-stage research included 1,234 Chinese subjects with CAD (22). In the discovery phase (total *N* = 896), the association of plasma metabolome and lipidome with immune cell-based inflammatory markers was first evaluated in the SII cohort (*N* = 892). Individuals with available data for low-grade inflammatory markers were then categorized into the INFLA cohort (*N* = 400) to investigate the association of plasma metabolome and lipidome with low-grade inflammatory markers. All these patients were consecutively enrolled from Guangdong Provincial People’s Hospital between 2010 and 2014. In the validation phase (total *N* = 338), 338 patients and 157 patients in the SII and INFLA cohorts, respectively, were enrolled from Guangdong Provincial People’s Hospital from 2017 to 2018.

All individuals were 18–80 years old and have met the diagnostic criteria for CAD. The exclusion criteria were as follows: (1) renal failure, serum creatinine >3.0 mg/dl, renal dialysis, or transplantation; (2) liver failure, alanine aminotransferase >135 U/L, or cirrhosis; (3) in pregnancy or lactation; (4) malignant tumor or hemodialysis; (5) WBC >20 × 10^9^/L; (6) autoimmune diseases; and (7) unavailability of inflammatory markers. Patients with hs-CRP or CRP ≥10 mg/L were not included in the INFLA cohort. Demographic information, medication history, and laboratory measurements were obtained from hospital electronic records.

This study was approved by the Medical Ethical Review Committee of Guangdong Provincial People’s Hospital and conducted in compliance with the Helsinki Declaration. Written informed consent was obtained from all the subjects.

### Measurements and Characterization of Inflammatory Markers

Fasting venous blood was collected upon admission and analyzed in accordance with the manufacturer’s instructions at the Laboratory Department of Guangdong Provincial People’s Hospital. Serum CRP levels were measured by immunonephelometric assay (IMMAGE; Beckman Coulter, USA). WBC, lymphocyte, neutrophil, and platelet counts were estimated using an automated blood cell counter (LH780; Beckman Coulter, Brea, CA, USA). Plasma fibrinogen levels were measured using a clot-based turbidimetric detection system.

NLR, PLR, and SII were calculated as the ratio of neutrophil count to lymphocyte count, the ratio of platelet count to lymphocyte count, and the ratio of neutrophil count multiplied by PLR, respectively (16). The INFLA score (11) captures serum and cellular circulating inflammatory markers and is composed of CRP, WBC, platelet count, and granulocyte-to-lymphocyte ratio, which was calculated based on 10 tiles of each biomarker level. The highest (7–10) and lowest (1–4) deciles were scored as positive and negative from 1 to 4, respectively, and deciles 5–6 were scored as zero. The INFLA score is the sum of the four biomarkers and ranges from -16 to 16.

### Atherosclerosis Evaluation *Via* Coronary Angiography and Carotid Artery Ultrasound

Coronary angiography and carotid artery ultrasonography were performed by expert sonographers following the standard protocol. Images of coronary angiograms were acquired using the Syngo Dynamics cardiovascular imaging software (Siemens Medical Solutions, United States, Inc., Malvern, PA, USA). The vascular lesion severity of CAD was mainly categorized as one vessel, two vessel, and three vessel or left main disease. Vessel disease was defined as ≥50% significant stenosis in major coronary arteries, including the left main coronary artery, left anterior descending artery, left circumflex artery, right coronary artery, or one of their major branches. The Gensini score was also used to characterize the atherosclerotic burden of CAD (23). Each identified lesion was scored based on the diameter stenosis (1 = 25%, 2 = 50%, 4 = 75%, 8 = 90%, 16 = 99%, and 32 = 100%). The score was then multiplied by a predefined factor on the basis of the functional importance of the lesion vascular segment of the lesion. Finally, the sum of scores was calculated for each patient.

An ATL HDI 3000 ultrasound system (Advanced Technology Laboratories, Bothell, WA, USA) was used to detect the carotid arteries. The mean and maximum values of the left and right intima–media thickness (IMT) were used for subsequent analyses.

### Plasma Sample Collection

Each eligible subject fasted overnight to minimize the influence of diet on the metabolite levels. Venous blood samples were collected in ethylene diamine tetraacetic acid vacutainer tubes in the morning and immediately cooled at 4°C. Plasma was separated within 2 h by centrifugation at 2,095 *g* and 4°C for 10 min and stored at −80°C until metabolomic analysis.

### Widely Targeted Metabolomic Analysis and Data Preprocessing

A full description of the widely targeted metabolomic analysis and data preprocessing (24) is provided in the **Supplementary Material** and **Supplementary Figure S1**. Metabolite molecules were extracted from the plasma samples and analyzed by an ultra-performance liquid chromatography and electrospray ionization–tandem mass spectrometry system in positive and negative ionization modes by Metware Biotechnology (Wuhan, China).

A total of 860 metabolites were identified, including nucleosides, hormones, carbohydrates, organic acids and derivatives, amino acids and derivatives, ceramide (Cer), cholesteryl esters, diacylglycerol, lysophosphatidic acid, phosphatidylcholines (PC), alkylphosphatidylcholine [PC(O)], lysophosphatidylcholine (LPC), lysophosphatidylethanolamine (LPE), lysophosphatidylserine (LPS), monoglyceride, phosphatidylethanolamine (PE), phosphatidylethanolamine [PE(P)], phosphatidic acid, phosphatidylserine, phosphatidylglycerol, and triacylglycerol (TG).

### Statistical Analysis

Among the clinical characteristics of the study populations, continuous variables were described using means ± standard deviation and were compared by *t*-test for normal distribution or Mann–Whitney *U*-test for non-normal distribution. Categorical variables were presented as counts (percentages) and were compared using chi-square tests. Statistical significance was defined as *P*-value <0.05.

Linear regression analysis in a stepwise regression (forward and backward) was used to examine the associations of metabolomic and lipidomic profiles with log-transformed inflammatory markers, including general systemic immune-inflammatory markers (NLR, PLR, and SII) in the SII cohort and low-grade inflammatory markers (hsCRP, WBC, FIB, and INFLA) in the INFLA cohort, adjusting for main potential confounders, including age, sex, smoking, hypertension, diabetes mellitus, arrhythmia, low-density lipoprotein cholesterol (LDLC), high-density lipoprotein cholesterol (HDLC), triglycerides (TRIG), aspartate aminotransferase (AST), estimated glomerular filtration rate (eGFR), and medications. Metabolites with a false discovery rate (FDR)-corrected *P*-value <0.05 were considered statistically significant. The same linear regression analysis was performed to assess the relationship between inflammation-associated metabolite markers and carotid (mean and maximum IMT) and coronary (severity of vascular lesion and Gensini score) atherosclerotic indicators. *P* value <0.05 was considered statistically significant.

To further investigate the association of metabolomic and lipidomic signatures with different inflammatory states, the participants were divided into two groups based on the median of the four main inflammatory markers. In the SII cohort, the inflammatory states of the two definitions were based on the median of SII and NLR, low *versus* (*vs*.) high SII states and low *vs*. high NLR states. In the INFLA cohort, inflammatory states included low *vs*. high INFLA states and low *vs*. high hs-CRP states. Least absolute shrinkage and selection operator (LASSO)-penalized logistic regression (“glmnet” package) was initially performed on 860 metabolites to avoid the multiple co-linear relationships of so many metabolic features by reducing the dimension of metabolites and screen important metabolites for different inflammatory states (low *vs*. high SII states, low *vs*. high NLR states, low *vs*. high INFLA states, and low *vs*. high hs-CRP states). In this analysis, the optimal tuning parameter *λ* was determined using 5-fold cross-validation with 200 iterations. Optimal lambda was adopted as minimum mean cross-validated error, namely, “min” lambda. The relative contribution of the features to the inflammatory states was assessed based on the occurrence frequency in multivariate training. A feature with an occurrence frequency of over 100 times was chosen as important metabolites for further analysis.

Furthermore, adjusted logistic regression in a stepwise regression (forward and backward) was conducted for the metabolites screened from LASSO against different inflammatory states to further identify individual metabolic markers independent of potential confounder to estimate the odds ratios (ORs) and 95% confidence intervals (CIs). The potential confounders were adjusted similarly to linear regression analysis. Statistical significance was defined as a *P*-value <0.05. Open database sources, including the Kyoto Encyclopedia of Genes and Genomes database (http://www.genome.jp/kegg/) and the MetaboAnalyst (https://www.metaboanalyst.ca) (version 5.0), were used to reveal the highly enriched metabolic pathways of the significant metabolites identified by LASSO. Statistical significance was set at *P <*0.05.

In addition to association testing, we also searched for a combination of metabolites with potential predictive values in discriminating different inflammatory states. For model development, a discriminative model was developed simply based on the important metabolites selected from LASSO by applying multivariate logistic regression analysis in a stepwise regression (forward and backward). The metabolites with *P <*0.05 were retained, and the model with minimal Akaike information criterion was recognized as the optimal model. A binary logistic regression model was then fitted based on the chosen metabolite markers to assess the discrimination probability of the models; a combined discriminating score (probability) was calculated using the formula = 1/1 + exp {-[intercept + coefficient1 (marker1) + coefficient2 (marker2) … + coefficient n (marker n)]}. The area under the curve (AUC) of the receiver operating characteristic (ROC) curve was used to determine the ability of the model to distinguish between different inflammatory states, with 1.0 indicating perfect discrimination. Differences in the combined scores between low and high inflammatory states in the validation cohort were evaluated using the Wilcoxon rank-sum test.

All statistical analyses were performed using R (version 4.1.0, http://www.R-project.org/).

## Results

### Characteristics of the Study Population

The clinical characteristics of the patients in the discovery phase are shown in **Table 1** and **Supplementary Table S1**. In the SII cohort (*N* = 892), the participants had an average age of 62.78 years, and 80% were male. Patients in the high median SII and NLR groups had hypertension, high PLR, hs-CRP, WBC, FIB, GLUC, mean IMT, max IMT, and Gensini score but low eGFR and HDLC. In the INFLA cohort (*N* = 400), the participants had an average age of 62.58 years, and 81% were male. The patients in the high median INFLA and hs-CRP groups exhibited high SII, WBC, FIB, and GLUC. Compared with chronic inflammatory markers, systemic immune-inflammatory indices were more important for increased atherosclerosis risk.

**Table 1:** Baseline characteristics of the population in the discovery phase.

Characteristics	SII cohort (*N* = 892)	INFLA cohort (*N* = 400)
Age, years	62.78 (10.06)	62.58 (9.78)
Male	714 (80.04)	325 (81.25)
SBP, mmHg	130.34 (18.70)	130.26 (18.85)
BMI, kg/m^2^	24.22 (3.15)	24.19 (3.12)
Current smokers	258 (29.22)	116 (29.22)
Comorbidities		
Arrhythmia	67 (7.51)	28 (7.00)
Diabetes mellitus	243 (27.24)	112 (28.00)
Hypertension	536 (60.09)	252 (63.00)
Laboratory data		
eGFR, ml/min/1.73m^2^	96.17 (78.65)	102.04 (103.77)
AST, U/L	26.71 (10.69)	25.78 (9.20)
GLUC, mmol/L	6.74 (2.70)	6.82 (2.84)
HDLC, mmol/L	0.96 (0.26)	1.01 (0.27)
LDLC, mmol/L	2.57 (0.90)	2.57 (0.90)
TRIG, mmol/L	1.62 (1.14)	1.64 (1.31)
Medications		
β-Blockers	797 (89.45)	363 (90.75)
CCBs	242 (27.16)	127 (31.75)
PPIs	426 (47.81)	211 (52.75)
Inflammatory makers		
SII	618.52 (489.65)	572.23 (450.39)
NLR	2.80 (1.85)	2.72 (1.92)
PLR	127.13 (60.46)	122.15 (55.97)
hs-CRP, mg/L	8.30 (17.07)	2.75 (2.47)
WBC, 10^9^/L	7.46 (2.04)	7.23 (1.89)
FIB, g/L	4.15 (1.29)	3.74 (0.94)
INFLA score	–	0.04 (5.87)
Atherosclerosis indicators		
Mean IMT, mm	0.95 (0.20)	0.93 (0.20)
Maximum IMT, mm	1.01 (0.25)	0.98 (0.22)
Gensini score	23.08 (12.03)	23.16 (12.51)
Severity of vascular lesion		
One-vessel disease	229 (27.59)	97 (26.43)
Two-vessel disease	260 (31.33)	110 (29.97)
Three-vessel or left main disease	341 (41.08)	160 (43.60)

Data are shown as mean (standard deviation) or n (%).

SBP, systolic blood pressure; BMI, body mass index; eGFR, estimated glomerular filtration rate; AST, aspartate aminotransferase; GLUC, glucose; HDLC, high-density lipoprotein cholesterol; LDLC, low-density lipoprotein cholesterol; TRIG, triacylglycerol; β-blockers, β receptor blockers; CCBs, calcium channel blockers; PPIs, proton pump inhibitors; SII, systemic immune-inflammation index; NLR, neutrophil–lymphocyte ratio; PLR, platelet–lymphocyte ratio; hs-CRP, high-sensitivity C-reactive protein; WBC, white blood cell count; FIB, fibrinogen; INFLA score, low-grade inflammation score; IMT, intima–media thickness.

The validation phase included 338 participants, and their baseline characteristics are summarized in **Supplementary Table S2**.

### Relationship Between Plasma Metabolite Profile and General Systemic Immune-Inflammatory Index

**Figure 2:**
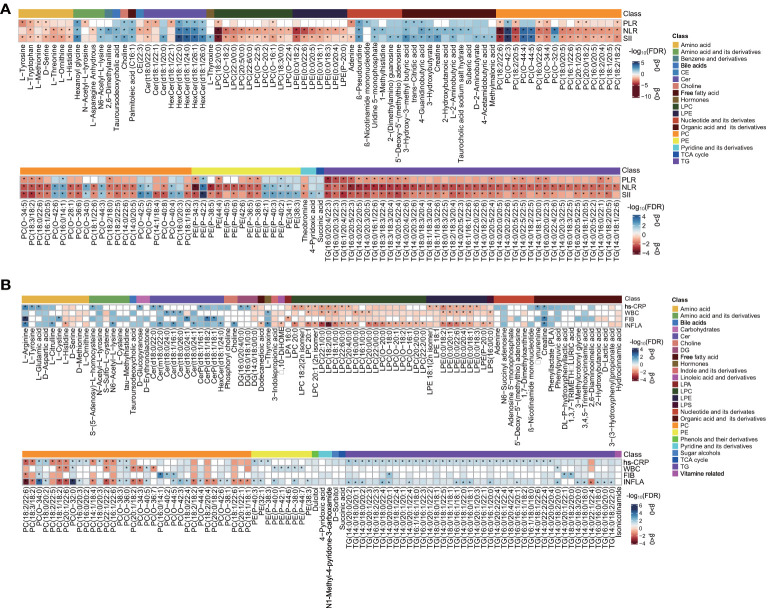
Assessment of metabolite associated with each inflammatory marker. **(A)** Heat map of lipid species [false discovery rate (FDR) <0.01] and metabolites (FDR < 0.05) and systemic immune-inflammatory markers in the SII cohort. **(B)** Heat map of lipid species (FDR < 0.05) and metabolites (FDR < 0.1) and low-grade inflammatory markers in the INFLA cohort. The blue- and red-color-coded FDR indicate positive and negative associations, respectively. Significance (FDR < 0.05) by linear regression analyses is marked with an asterisk, adjusted for age, sex, smoking, hypertension, diabetes mellitus, arrhythmia, LDLC, HDLC, TRIG, AST, eGFR, and medications. CE, cholesteryl esters; Cer, ceramide; HexCer, hexosylceramide; CerP, ceramide phosphate; LPC; lysophosphatidylcholine; LPE, lysophosphatidylethanolamine; PC, phosphatidylcholine; PE, phosphatidylethanolamine; TCA, tricarboxylic acid cycle; TG, triacylglycerol; DG, diacylglycerol; LPA, lysophosphatidic acid; LPS, lysophosphatidylserine. The other abbreviations are as listed in **Table 1**. The corresponding beta estimates and FDR values are given in **Supplementary Tables S3**, **S5**.

A linear regression analysis of metabolites in relation to the immune cell-based inflammatory index (SII, NLR, and PLR) was conducted, accounting for the 12 main confounders. SII, NLR, and PLR showed significant associations (FDR < 0.05) with 170, 204, and 84 out of 860 plasma metabolites, respectively (**Figure 2A** and **Supplementary Table S3**). Among the 55 metabolites common to the three inflammatory markers (**Supplementary Figure S2A**), several lipids (*e*.*g*., Cer, LPC, PC, PE, and TG), L-tryptophan, and the D-serine levels were negatively associated, and HexCer, choline, β-pseudouridine, 4-guanidinobutyric acid, hexanoyl glycine, and 3-hydroxy-3-methyl butyric acid levels were positively associated. In addition, LPC (18:2/0:0) and PC (18:2/22:6) showed a strong (FDR < 1E-04) negative association, and PC (O-42:3) showed a strong positive correlation to NLR and SII. PE(P-42:2) and HexCer (d18:1/22:0, d18:1/24:0) were strongly positively associated with SII, and PC (18:2/20:5) was strongly negatively associated with NLR. Only 61 significant metabolites were shared by NLR and SII. Except for the aforementioned lipid species, LPE (0:0/18:2, 0:0/22:6), L-threonine, L-ornithine, and L-histidine were negatively related, and 3-hydroxybutyrate, tauroursodeoxycholic acid, L-2-aminobutyric acid, and uridine 5-monophosphate were positively related. For the metabolites uniquely common to PLR and SII, seven significant metabolites exhibited adverse associations (L-tyrosine, L-methionine, and adenine) and positive associations (PC 18:1/20:1, PC 18:1/22:4, trans-citridic acid, and Cer d18:0/24:1).

**Figure 3:**
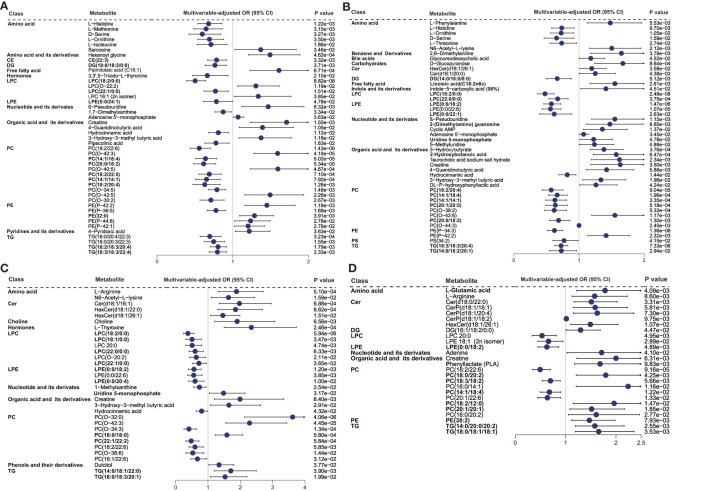
Relationship between metabolic signatures and different inflammatory states. Forest plot of odds ratios (OR) and 95% confidence intervals (95% CI) for the logistic regression of individual metabolites/lipid species associated with inflammatory states of high *vs*. low **(A)** SII and **(B)** NLR in the SII cohort and high *vs*. low **(C)** INFLA and **(D)** hs-CRP in the INFLA cohort, adjusted for age, sex, smoking, hypertension, diabetes mellitus, arrhythmia, LDLC, HDLC, TRIG, AST, eGFR, and medications (*P* < 0.05). The abbreviations are as in **Figure 2**.

The patients were categorized into two levels of inflammatory states based on median SII or NLR for LASSO logistic regression analysis (**Supplementary Table S4**). Sixty-seven metabolites were screened out with an occurrence frequency of more than 100 times in the classification of low *vs*. high SII and NLR states, including 24 overlapping metabolites. The logistic regression analysis adjusted for clinical confounders showed that 45 (**Figure 3A**) and 46 (**Figure 3B**) metabolites were still significant with the SII and NLR states (*P* < 0.05), respectively. β-Pseudouridine, PE (P-42:2), creatine, 4-guanidinobutyric acid, 3-hydroxy-3-methyl butyric acid, and adenosine 5′-monophosphate showed positive associations with SII and NLR states; LPC (18:2/0:0), PC (18:2/20:4, 14:1/18:4, 14:1/14:1), amino acids (L-histidine, L-ornithine, and D-serine), and hydrocinnamic acid exhibited negative associations. For the metabolites uniquely related to SII states, positive associations were observed for PCs (O-42:3, O-40:5, O-42:5), PEs (P-42:1, 32:0), hexanoyl glycine, sarcosine, and 4-pyridoxic acid. Inverse associations were found for a wealth of lipids (LPC 22:1/0:0, PCs, and TGs), amino acids (L-methionine and L-isoleucine), pipecolinic acid, 3,3′,5-triiodo-L-thyronine, and 1,7-dimethylxanthine. For the metabolites unique to NLR states, 3-hydroxybutyrate, 2-hydroxybutanoic acid, taurocholic acid sodium salt hydrate, L-phenylalanine, 2-(dimethylamino) guanosine, DL-P-hydroxyphenyllactic acid, Cer (d18:1/20:0), HexCer(d18:1/26:1), and indole-5-carboxylic acid showed positive associations, and PE(P-34:3), LPE (0:0/18:2, 0:0/22:6, 0:0/22:1), LPC (22:0/0:0), PC (20:1/20:5, O-38:2, 20:0/18:2), and L-threonine exhibited adverse associations.

### Relationship Between Plasma Metabolite Profile and Low-Grade Inflammatory Index

Four main low-grade inflammatory markers (INFLA, hs-CRP, WBC, and FIB) were analyzed in the INFLA cohort and showed significant associations (FDR < 0.05) with 89, 80, 32, and 15 metabolites, respectively, after the adjustment for 12 main confounders (**Figure 2B** and **Supplementary Table S5**). None of the metabolites were common to all inflammatory markers (**Supplementary Figure S2B**), and eight metabolites were nominally significant (FDR < 0.1) for all four markers, mainly including the positive associations of PC (O-34:0, O-32:0, O-36:0) and creatine and the adverse associations of LPE (0:0/18:2), LPC (18:2/0:0, 18:1/0:0), and PC (18:1/18:2). There were 19 (FDR < 0.05) and 38 (FDR < 0.1) metabolites shared by at least three of the assessed inflammatory traits. Positive associations with PC (O-38:3, O-38:1) and TG (14:0/18:2/20:0) and inverse associations with LPC (*e*.*g*., 20:1, 18:1/0:0), LPE (*e*.*g*., 0:0/18:2, 0:0/20:1), and PC (*e*.*g*., 20:1/22:6, 22:1/22:2) were shared by INFLA, WBC, and hs-CRP (FDR < 0.1). Besides these, several TGs (*e*.*g*., 14:0/20:0/20:2, 18:0/18:0/18:1), CerP (d18:1/16:1, d18:1/18:2), and amino acids (L-arginine, D-aspartic acid, L-citrulline) were positively associated with hs-CRP, FIB, and INFLA (FDR < 0.1), and LPC (*e*.*g*.,18:2/0:0, 18:1/0:0) and PCs (*e*.*g*., 18:2/22:6, 18:3/18:2) were negatively associated. Phenylpyruvic acid (positive) was shared by hs-CRP, WBC, and FIB; Cer (t18:0/26:0), PC (O-44:4), and L-thyroxine (positive) were shared by INFLA, WBC, and FIB (FDR < 0.1).

The patients were further divided into two levels of inflammatory state and underwent LASSO analysis (**Supplementary Table S6**). In the classification of low *vs*. high INFLA and hs-CRP states, 47 and 30 metabolites showed an occurrence frequency of more than 100 times, respectively, and eight metabolites were identical. The metabolites chosen from LASSO were inputted into a logistic regression analysis with adjustment for potential confounders. The results showed that 32 (**Figure 3C**) and 26 (**Figure 3D**) metabolites remained significant with INFLA and hs-CRP states (*P* < 0.05), respectively. In particular, negative associations with INFLA and hs-CRP states were found for PC (18:2/22:6), LPE (0:0/18:2), and LPC 20:0, and positive associations were observed for creatine, L-arginine, and HexCer (d18:1/26:1). For the metabolites unique to INFLA states, positive associations were found for lipids, such as PCs (O-32:0, O-42:3, 18:0/18:0), Cer (d18:1/16:1), HexCer (d18:1/22:0), TG (14:0/18:1/22:0, 18:0/18:3/20:1), L-thyroxine, choline, N6-acetyl-L-lysine, 3-hydroxy-3-methyl butyric acid, uridine 5-monophosphate, and dulcitol, and inverse associations were observed for many lipids (LPC, *e*.*g*., 18:2/0:0, 18:1/0:0; PC, *e*.*g*., O-34:3, 22:1/22:2; and LPE, *e*.*g*., 0:0/22:6, 0:0/20:4), hydrocinnamic acid, and 1-methylxanthine. For the metabolites unique to hs-CRP states, only three PCs (18:3/18:2, 14:1/18:4, 20:1/22:6) were associated with low hs-CRP states, and TG (14:0/20:0/20:2, 18:0/18:1/18:1), Cer (d18:0/22:0), CerP (d18:1/16:1, d18:1/20:4), PC (e.g., 18:0/20:2, 16:0/14:1), L-glutamic acid, and phenyllactate were associated with high hs-CRP states.

### Pathway Analysis

**Figure 4:**
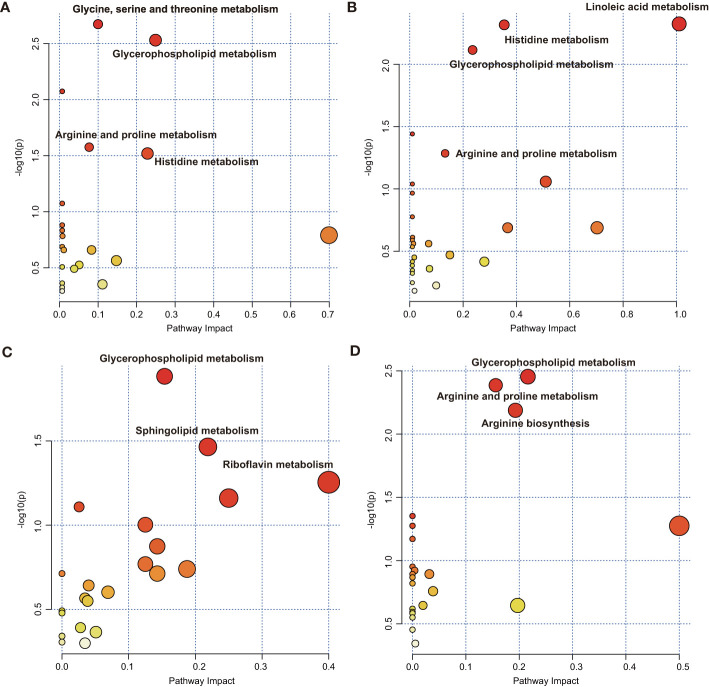
Metaboanalyst pathway analysis showing disturbed pathways for different inflammatory states. High *vs*. low **(A)** SII and **(B)** NLR in the SII cohort and high *vs*. low **(C)** INFLA and **(D)** hs-CRP in the INFLA cohort. The abbreviations are as in **Figure 1**.

Pathway metabolomics analysis was conducted to identify the most relevant pathways involved in inflammatory conditions by employing the metabolic markers from LASSO (**Supplementary Table S7**). The results showed that glycine, serine, and threonine metabolism, glycerophospholipid metabolism, arginine and proline metabolism, and histidine metabolism, with pathway impact values of 0.09, 0.24, 0.07, and 0.22, respectively, were altered in the plasma of patients with CAD with elevated SII states (*P* < 0.05) (**Figure 4A**). Glycerophospholipid metabolism (impact = 0.23) and histidine metabolism (impact = 0.34) were the key perturbed pathways with elevated NLR states (**Figure 4B**), and arginine and proline metabolism was nominally significant with elevated NLR states (impact = 0.12, *P* = 0.052). Linoleic acid metabolism was the most relevant pathway in NLR states (impact = 1, *P* = 0.005). Target pathways that were altered in high INFLA states (**Figure 4C**), including glycerophospholipid metabolism with an impact of 0.15, sphingolipid metabolism with an impact of 0.22, and riboflavin metabolism, showed nominal relevance (impact = 0.4, *P* = 0.056). Glycerophospholipid metabolism (impact = 0.22), arginine and proline metabolism (impact = 0.16), and arginine biosynthesis (impact = 0.19) were the key pathways associated with high hs-CRP states in patients with CAD (**Figure 4D**). These results suggested that perturbed glycerophospholipid metabolism and arginine and proline metabolism are the key metabolic characteristics of patients with CAD and who have elevated immune-inflammatory states.

### Predicting an Advanced Inflammatory State by Metabolites

**Figure 5:**
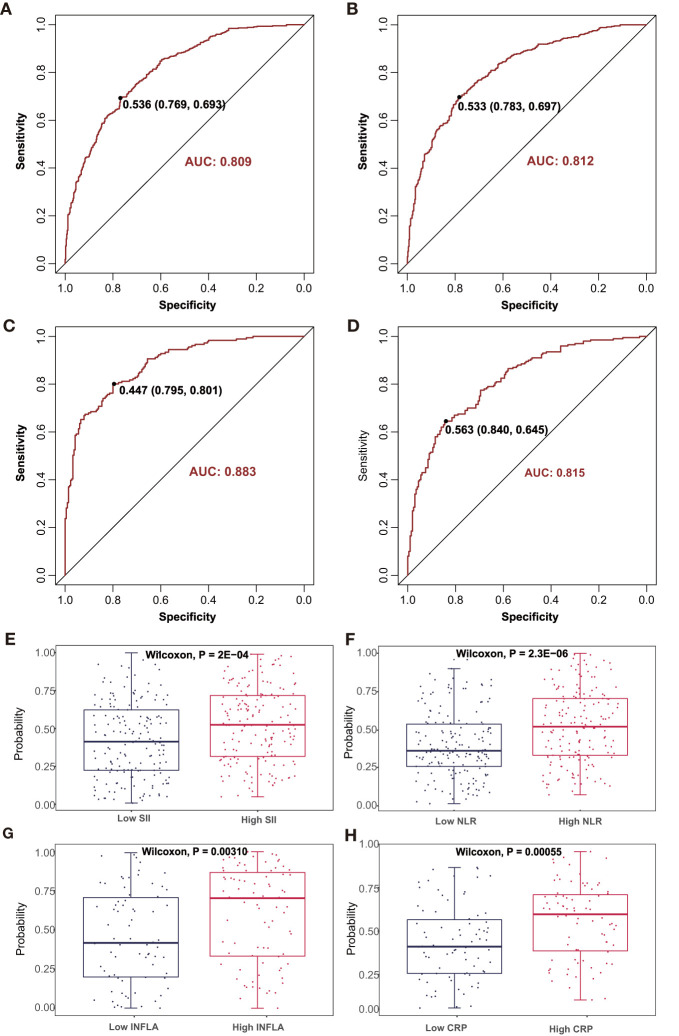
The discriminating performance in the discovery phase is shown by ROC curves between high *vs*. low **(A)** SII and **(B)** NLR in SII cohort 1 and high *vs*. low **(C)** INFLA and **(D)** hs-CRP in INFLA cohort 1. The combined discriminative score in the validation phase was compared between high *vs*. low **(E)** SII and **(F)** NLR in SII cohort 2 and high *vs*. low **(G)** INFLA and **(H)** hs-CRP in INFLA cohort 2. AUC, area under curve; ROC, receiver operating characteristic. The other abbreviations are as in **Figure 1**.

A discriminative combination of the aforementioned selected metabolites from LASSO to predict an advanced inflammatory state was searched among patients with CAD (**Supplementary Table S8** and **Figure 5**). A stepwise multivariate logistic regression analysis based on the metabolic markers identified by the LASSO analysis was further applied for model development. Finally, a model with 29 metabolites was obtained to predict the SII states; its classification was moderately robust as indicated by an AUC of 0.81 (**Figure 5A**). When the 33 metabolites retained in the multivariate logistic training were applied for predicting the NLR states, the AUC score was 0.81 (**Figure 5B**). Furthermore, a robust classification was achieved for INFLA states with an AUC of 0.88 by using a subset of 24 metabolites (**Figure 5C**). The model containing 15 metabolites for discriminating hs-CRP states also obtained a modestly good performance with an AUC of 0.82 (**Figure 5D**). The established model was subsequently assessed to determine the probability for differentiating the four types of inflammatory states in the validation cohort (**Supplementary Table S2**). The validation cohort was also divided into two types of inflammatory study cohorts: the SII cohort (*n* = 338) and low-grade inflammatory cohort (*n* = 157). The combined diagnostic score was used to reveal the potential ability to discriminate inflammatory states to differentiate patients with low *versus* high inflammatory states for SII (*P* = 0.0002, **Figure 5E**), NLR (*P* < 0.0001, **Figure 5F**), INFLA (*P* = 0.0031, **Figure 5G**), and CRP (*P* = 0.0006, **Figure 5H**).

### Relationship of Inflammation-Associated Metabolites With Atherosclerosis Indicators

**Figure 6:**
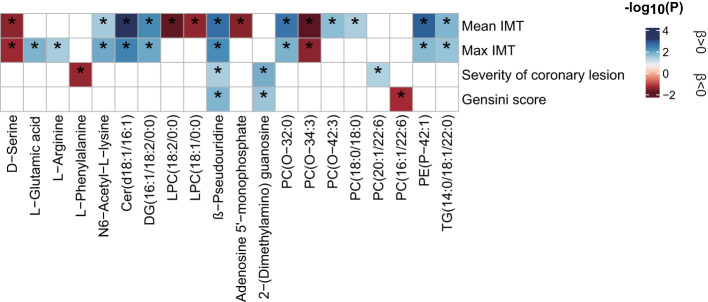
Relationship between inflammatory state-associated metabolites and atherosclerosis indicators. The blue- and red-color-coded *P*-values indicate positive and negative associations, respectively. Significance (*P* < 0.05) by linear regression analyses is marked with an asterisk. All analyses were adjusted for 12 main confounders. The corresponding beta estimates and *P*-values are given in **Supplementary Table S9**. IMT, intima-media thickness.

Linear regression analysis was performed to examine the relationship between the inflammation-associated metabolites and atherosclerosis indicators (*P* < 0.05, **Figure 6** and **Supplementary Table S9**). The results showed that the increased levels of Cer (d18:1/16:1), β-pseudouridine, PE(P-42:1), DG (16:1/18:2/0:0), PC (O-32:0), TG (14:0/18:1/22:0), and N6-acetyl-L-lysine were associated with elevated atherogenesis as manifested by the high indicators of arteriosclerosis plaque formation (mean IMT and max IMT). Meanwhile, PC (O-34:3) and D-serine were associated with decreased atherogenesis. Positive correlations for PC (O-42:3, 18:0/18:0) and mean IMT, and L-glutamic acid, L-arginine, and max IMT and inverse correlations for LPC (18:2/0:0, 18:1/0:0) and mean IMT were also found. A further analysis was conducted on the indicators of coronary arteriosclerosis lesions. We observed that 2-(dimethylamino) guanosine, β-pseudouridine, and PC (20:1/22:6) contributed to the increased severity of vascular lesions, whereas L-phenylalanine was associated with decreased severity. Similarly, 2-(dimethylamino) guanosine and β-pseudouridine were also found to be associated with high Gensini scores, whereas PC (16:1/22:6) was associated with low Gensini scores. In particular, β-pseudouridine was the only metabolite that was consistently shared by the four arteriosclerosis traits.

## Discussion

This study consolidated that immune-inflammatory processes involve the majority of metabolic signature changes in patients with CAD. The results revealed 45 and 46 metabolites significantly associated with general SII and NLR states, and 32 and 26 metabolites were significantly associated with chronic INFLA and hs-CRP states, respectively. The combinations of metabolite markers could be used to discriminate elevated inflammatory states well. A pathway analysis indicated that glycerophospholipid metabolism and arginine and proline metabolism were the key altered metabolic pathways for both general systemic immune and low-grade inflammation states. Finally, 20 inflammation-associated metabolites [*e*.*g*., Cer (d18:1/16:1) and β-pseudouridine] contributed to increased arteriosclerosis, and β-pseudouridine was the only metabolite that contributed to both carotid and coronary arteriosclerosis indicators. In summary, this study provides novel insights into altered molecular patterns, potentially helping to understand the adverse effects of inflammatory processes on metabolic disturbances and arteriosclerosis.

The results specially showed that glycerophospholipid metabolism was the major pathway involved in both general systemic-immune and low-grade inflammatory states, suggesting that phospholipids are potential inflammatory mediators. Our results are similar to previous findings (20, 25). Most PCs or PEs are negatively associated with the inflammatory index, especially those with more polyunsaturated acyl chains (*e*.*g*., PC 18:2/20:4, 18:2/22:5; PE P-36:5). Inversely, those with lower polyunsaturated acyl chains (*e*.*g*., PE 32:0; PC O-32:0, 18:0/18:0, 16:0/14:1) tend to be positively associated. However, not all PC or PE species followed this rule; those with very long chain or that have a lot of double bond (*e*.*g*., PC O-42:3, O-42:6; PE P-44:8) were instead positively associated. PC or PE containing saturated acyl chains (*e*.*g*., PC O−32:0, 18:0/18:0) and very long or polyunsaturated acyl chains (*e*.*g*., PC O−42:3, 20:1/22:6; PE P−42:1) also contributed to atherosclerosis, whereas those containing polyunsaturated fatty acids (PC O−34:3, 16:1/22:6) decreased with atherogenesis. These findings were partly supported by previous studies (26, 27). By and large, the functional properties of different PC species depend on the composition of the unsaturated acyl chains (28). PCs or PEs with an extremely long chain and are polyunsaturated have protective effects in the inflammatory process and atherosclerosis, but those that are highly saturated have detrimental effects (27, 29). The shorter and highly saturated acyl chains of lipid species allow less fluidity of the lipid monolayer, hence affecting the ability of high-density lipoproteins to uptake cholesterol from peripheral tissues and phospholipid hydroperoxides from low-density lipoproteins (LDL) (30, 31). PE(P) and PE (O), known as plasmalogens, have a high proportion of polyunsaturated alkenyl linked fatty acids and are crucial in storing precursors as inflammatory mediators, regulation of membrane fluidity, and anti-oxidation (32). However, it is not clear whether phospholipids containing very long or polyunsaturated acyl chains are oxidated to produce harmful lipid components or lead to cell membrane instability in the course of heightened inflammation and atherosclerosis.

LPC and LPE are primarily catalyzed by phospholipase A1 or A2 from PC and PE. We observed that most LPC and LPE were negatively associated with inflammatory markers. LPC (18:1, 18:2) was associated with decreased carotid atherosclerosis. Previous studies have also found that LPC 18:1 and 18:2 and LPE were negatively associated with hs-CRP levels (20) or CVD risk (33, 34). However, it was thought that phospholipase catalysis is intensified under inflammation and promotes the inflammatory process (35). Increased phospholipase catalysis could promote arteriosclerosis by polarizing macrophage activation toward the M1 phenotype (36). LPC acts as an important mediator of endothelial cell activation (37) and inflammation (38). This controversial finding could be explained as an increase of their catabolism or removal from the blood into the tissues during advanced inflammatory states and severe endothelial dysfunction in arteriosclerosis in the form of modified lipoprotein or albumin (39).

In addition to glycerophospholipids, we observed interesting associations with ceramides and TGs. Collectively, we observed a positive association with ceramides; HexCer (d18:1/22:0, d18:1/26:1), Cer (e.*g*., d18:1/16:1, d18:0/22:0), and Cer P (*e*.*g*., d18:1/16:1) were positively associated with INFLA or hs-CRP or NLR. Cer (d18:1/16:1) was also positively related to carotid atherosclerosis. Consistent with our findings, Cers have been observed with pleiotropic pro-inflammatory effects. Increased levels of total HexCers and Cers were found in the serum of RA patients (40). Plasma ceramides are closely associated with increased levels of circulating inflammatory cytokines in coronary disease (41) and are independent biomarkers of CVD (42). Different Cers were found with different effects on CVD (42); long-chain species (d18:1/18:0) were more harmful than very-long-chain (d18:1/24:0) species. Cers are known to fuel the atherosclerosis process *via* inducing the accumulation of lipoproteins, driving transcytosis of oxidized LDL into the blood vascular walls, and monocyte adhesion in vessel walls (43, 44). However, it was reported that the associations between C16:0 and C24:1 ceramides and carotid artery atherosclerosis may involve inflammation and immune activation rather than LDL (45). Cers regulate immune response and the inflammatory process *via* activation of many stress, such as the NFκB signaling pathway (46). Interestingly, TGs exhibited the opposite effects in two types of inflammatory indices; several polyunsaturated fatty acid-containing TGs (*e*.*g*., 16:0/20:4/22:3, 14:0/22:0/22:6) were negatively associated with SII or NLR; other TGs with more saturated and monosaturated fatty acids (*e*.*g*., 14:0/18:1/22:0, 16:0/16:1/18:1) are positively associated with INFLA and hs-CRP. Moreover, TG (14:0/18:1/22:0) is also positively correlated with carotid atherosclerosis. The opposite effects that we observed were also following the rules that TGs of lower carbon and unsaturation tended to be positive, whereas polyunsaturated TGs tend to be negatively associated with CVD risks (47). The harmful effects of saturation in TGs may be due to the higher pro-atherogenic potential of saturated fats by promoting lipoprotein accumulation in the vessel wall, foam cell formation, and proinflammatory and proatherogenic protein (48, 49). Different stages of biological processes, such as initiating *versus* resolving inflammatory responses, would lead to diverse associations with lipid species. Thus, only a deeper comprehension of the determinants between structure and functional properties in the different pathological contexts will allow them to be possibly effective pathogenetic targets of therapeutic strategies in CAD.

Importantly, amino acid (AA) metabolism is a critical regulator in inflammation and atherosclerosis. We found that arginine and proline metabolism was the key relevant pathway for SII and hs-CRP; histidine metabolism was key for SII and NLR states. The strongest associations with different inflammatory states were identified as L-arginine (positive) and L-histidine (negative). L-Arginine (L-Arg) also contributed to atherosclerosis indicators. Similarly, arginine and histidine have the greatest close relationship with inflammation and oxidative stress in obese women (50). L-Arg is a key regulator in the urea cycle, which has shown important roles in the inflammatory process (20, 21). Most importantly, L-Arg, as the only precursor for nitric oxide (NO), has been shown to alleviate the inflammatory process in CVD (51). NO is an important modulator of vascular endothelium function against atherosclerosis (52) *via* vascular vasodilation, platelet aggregation, and smooth muscle proliferation inhibition (53), whereas our findings may represent a deficiency in NO production *via* L-Arg. During advanced atherosclerosis, the NO synthase expression in endothelial cells decreases, and uncoupling leads to limiting NO production. The elevated reactive oxygen species promote NO and ${\text{O}}_{2}^{-}$ to form peroxynitrite, thereby resulting in endothelial dysfunction (54). Thus, it is still controversial in the benefit of L-Arg supplementation in advanced atherosclerosis (55). Besides this, L-Arg could be metabolized into creatine, guanidino compounds, and L-ornithine. L-Ornithine, in turn, could be a precursor of L-Arg synthesis. In particular, arginase, as part of the urea cycle, was an important regulator of NO production by competing for L-Arg to form L-ornithine (56). Increased arginase expression was stimulated by pro-inflammatory factors and advanced atherosclerosis. L-Ornithine was also a precursor of glutamate and polyamines (57). So, we observed that L-ornithine and L-glutamate were negatively and positively associated with inflammatory markers, respectively, which may be due to the increased synthesis of L-Arg and L-ornithine metabolism under oxidative stress. Moreover, we found that L-glutamate also contributed to increased atherosclerosis indicators. Consistent with our findings, a recent study reported that elevated blood glutamate levels are associated with increased IMT and may be partly attributed to IL-6-associated subclinical inflammation (58). Although the potential mechanisms of the adverse effects of L-glutamate on CVD are less clear, it was claimed that plasma glutamate may be involved in the pathogenesis of cardiometabolic diseases (59). Other L-Arg-derived compounds, including creatine and 4-guanidinobutyrate, showed similar relationships with inflammatory markers as L-Arg. Creatine is mainly stored in the skeletal muscle for energy metabolism; creatine generation from L-Arg may reduce the pool of L-Arg available for NO generation (60). 4-Guanidinobutyrate could regulate T cell proliferation and has a pro-inflammatory function (61). Therefore, further investigations are necessary to find the mechanism to maintain the balance of the L-Arg–nitric oxide pathway during advanced atherosclerosis to provide a promising strategy to tackle cardiovascular issues.

Consistent with our findings, histidine level was negatively related to indicators of systemic inflammation, such as NLR, IL-6, and CRP, in patients with colorectal cancer (62). Histidine is a recognized free radical scavenger. Histidine can suppress inflammation by inhibiting pro-inflammatory cytokine production, possibly *via* the NF-κB involved pathway, in human coronary arterial endothelial cells (63) and adipocytes (64). D-Serine was negatively correlated with SII and NLR and carotid artery atherosclerosis. Serine exerts beneficial effects of anti-inflammatory and antioxidative abilities by controlling glutathione synthesis and AMPK activation (65). D-Serine has been proposed as a treatment for psychiatric and neurological conditions (66) and could help vasodilation dependent on regulation of endothelial NO synthase activity. While the role of D-serine in cardiovascular disease was not exactly known, it is worth for further study. An AA derivative, N6-acetyl-L-lysine, was positively associated with NLR, SII, and INFLA and contributed to increased carotid atherosclerosis. N6-acetyl-L-lysine, an acetylation derivative of lysine, is involved in post-translational modification and regulates the function of crucial immune regulators such as the NF-κB family (67). Lysine acetyltransferases and lysine deacetylases (HDACs) are crucial enzymes in the regulation of lysine acetylation levels; they were a possible target for therapeutic interventions in CVD (68). Moreover, treatment of atherosclerosis-prone mice with the HDAC inhibitor increased the TNF expression and aggravated the neointimal lesions in arteries (69). The anti-atherosclerotic effects of HDAC sirtuin1 are possibly by regulation of eNOS activation (70). Therefore, future studies examining the impact of lysine acetylation on inflammation and atherosclerosis should help to further elucidate the potential of lysine acetylation as an attractive therapeutic target of CAD.

Except for the predominant role of lipid metabolism and amino acid metabolism, there were still additional metabolites worth paying attention to. The most important were two modified nucleosides, namely, 2-dimethylguanosine and pseudouridine, which were positively associated with NLR and SII and increased atherosclerosis indicators. To our knowledge, this study is the first to report that pseudouridine contributed to carotid and coronary atherosclerosis. The 2-dimethylguanosine and pseudouridine levels are elevated during oxidative stress in patients with pneumonia, sepsis, cancer, and CVD (71–73). 2-Dimethylguanosine is a primary tRNA-specific modified nucleoside that could reflect vascular cell stress and hyperproliferation in pulmonary arterial hypertension (73). Pseudouridine is most abundant in both rRNA and tRNA. Stress conditions may induce the enzymatic posttranscriptional modification in RNA, leading to the elevated release of modified nucleosides (74). In turn, RNA modification can regulate ribosome diversity and mRNA stability and facilitate protein synthesis (75), which may drive the translation of disease-related proteins. Thus, circulating or urinary pseudouridine is widely considered a marker of RNA degradation and cellular protein breakdown. Pseudouridine could stimulate the expression of inflammatory cytokines, including TNFα and IL1β, in monocytes and promote the inflammatory cascade by acting as damage-associated molecular patterns (71). Although there is no direct evidence in atherosclerosis, RNA modification was recognized as a possible therapeutic tool for CVD (76), especially N6-adenosine methylation, which can affect the progression of atherosclerosis by affecting endothelial dysfunction, cholesterol efflux, and monocyte aggregation. Therefore, we believe that 2-dimethylguanosine and pseudouridine may be good markers to reflect excessively damaging stress in vascular endothelial cells and poor health status during CAD progression. In addition, a polyphenolic compound, hydrocinnamic acid, could be anti-inflammatory by suppressing macrophage infiltration and NFκB activation in adipocytes and has various beneficial effects against CVD (77).

Metabolic flexibility varies in different inflammatory statuses (78). In acute or chronic inflammatory responses, different inflammatory markers would reflect distinct physiological roles at different stages, especially during disease progression, thus leading to different metabolic profile associations. The results also showed that SII and NLR are associated with multiple atherosclerosis indicators (5). Previous studies have mainly focused on CRP (20, 21) and neglected other important immune-inflammatory markers. Here the metabolic profile associated with the inflammatory index was similar to the findings in RA but not in healthy participants, thus suggesting the importance of revealing metabolic signatures under different disease conditions. Although the mechanism underlying the association of metabolites with unique parameters remains difficult to explain, the current results provide an opportunity to realize the difference in metabolic profiles for the inflammatory status defined by distinct inflammatory markers or indices important for CVD progression. Several limitations also should be considered. First, due to analytical platform upgrades and technical advances in mass spectrometry, the numbers of metabolites and lipid species detected were not completely the same, thereby resulting in the lack of several predictors for inflammatory models that might have affected the model estimation in the validation cohort. Second, this study has a cross-sectional, observational design. It is hard to obtain causal conclusions and exact mechanical links between metabolic markers and inflammation. Further interventional and prospective studies are necessary to confirm the causal effect. Finally, this study focused on baseline immune cell-based inflammatory and low-grade inflammatory markers. Future studies are needed to investigate the dynamic metabolic profiling of other established inflammatory factors, such as TNF-α, IL-1β, and IL-6, to expand the current findings to different inflammatory stages.

## Conclusions

This study is the first to examine the relationship between metabolic and lipid profiles and clinically important immune-inflammatory parameters among patients with CAD. The results showed that immune inflammation involves various metabolic changes in plasma metabolites and lipid species in patients with CAD. Metabolite combinations could discriminate elevated inflammatory states well. Glycerophospholipid metabolism and arginine and proline metabolism play predominant roles in heightened immune inflammation. These findings may aid in predicting different inflammatory states and developing new therapeutic strategies, such as novel targets of immunosuppressive AA-metabolizing enzymes, or mRNA modifications to regulate inflammation progression during CAD.

## Data Availability Statement

The primary data presented in this study are provided in the **Supplementary Material** (**Supplementary Table S10**).

## Ethics Statement

The studies involving human participants were reviewed and approved by the Medical Ethical Review Committee of Guangdong Provincial People’s Hospital. The patients/participants provided their written informed consent to participate in this study.

## Author Contributions

SZ was the principal investigator of this study and designed the study. QZ performed the data analysis and drafted the manuscript. YW and JMa assisted in statistical analysis and critically revised the manuscript. GG and JMe were responsible for patient recruitment and clinical data collection. XF, XC and CL assisted in data curation and revised the manuscript. All authors reviewed and approved the final manuscript.

## Funding

This work was funded by grants from the National Natural Science Foundation of China (no. 81872934) and the Key Area Research and Development Program of Guangdong Province, China (no. 2019B020229003). The funders were not involved in designing the study; collecting, analyzing, or interpreting the data; or writing or submitting the manuscript for publication.

## Conflict of Interest

The authors declare that the research was conducted in the absence of any commercial or financial relationships that could be construed as a potential conflict of interest.

## Publisher’s Note

All claims expressed in this article are solely those of the authors and do not necessarily represent those of their affiliated organizations, or those of the publisher, the editors and the reviewers. Any product that may be evaluated in this article, or claim that may be made by its manufacturer, is not guaranteed or endorsed by the publisher.
