# Supplementary material for: Comprehensive Metabolic Profiling of Inflammation Indicated Key Roles of Glycerophospholipid and Arginine Metabolism in Coronary Artery Disease
Source: Front Immunol. 2022 Mar 8;13:829425. doi: 10.3389/fimmu.2022.829425 (PMC8965586; doi:10.3389/fimmu.2022.829425)
Supplement: Supplementary file 1 [file DataSheet_1.docx]

Supplementary Material

Comprehensive Metabolic Profiling of Inflammation Indicated Key Roles of Glycerophospholipid and Arginine Metabolism in Coronary Artery Disease

**Qian Zhu^1, 2^, Yonglin Wu^3^, Jinxia Mai^3^, Gongjie Guo^1,2^, Jinxiu Meng^4^, Xianhong Fang^5^, Xiaoping Chen^6^, Chen Liu^7^, Shilong Zhong ^2, 1, 3, 4^***

***Correspondence:**

Shi-Long Zhong: [gdph_zhongsl@gd.gov.cn](mailto:gdph_zhongsl@gd.gov.cn)

# Supplementary Methods

**1.1 Sample preparation and extraction**

For metabolic compounds profiling, plasma sample was thawed on ice, 150 μL chilled methanol was added to 50 μL of each plasma for protein precipitation. The mixture was vortexed for 3 min and then centrifuged with 12,000 rpm at 4 ℃ for 10 min. The supernatant was transferred into another clean EP tube and centrifuged again for 5 min under the same conditions as the first time, and the supernatant after the second centrifugation was used for the following UPLC-MS/MS analysis.

For lipids profiling, the thawed sample was gently vortexed for 10s, and then centrifuged with 3000 rpm at 4 ℃ for 5 min. 50μL of each sample was homogenized with 1mL mixture (include methanol, MTBE and internal standard mixture), vortexed for 2 min, followed by the addition of 500 μL pure water, vortexed for 1min again, then centrifuged with 12,000 rpm at 4℃ for 10 min. 500 μL of supernatant was removed and dried. The residue was reconstituted in 100 μL of mobile phase B (seen as UPLC Conditions) and subsequently analyzed by UPLC-MS/MS system.

**1.2 UPLC Conditions**

Chromatographic analysis was performed using UPLC system (Shim-pack UFLC SHIMADZU CBM30A). For metabolic profiling (hydrophilic compounds), chromatographic separation was performed on the Waters ACQUITY UPLC HSS T3 reversed-phase column(1.8μm, 2.1mm×100mm) with the mobile phases A of 0.04% acetic acid (Fisher Scientific) in water (Millipore) (*v/v*) and B of 0.04% acetic acid in acetonitrile (Fisher Scientific) (*v/v*) at column temperature of 40℃ and gradient flow rate of 0.4 mL/min. Gradient elution was programmed as follows: 0-11.0 min increased from 5% to 95% B and maintained at 95% B for 1min,12-12.1 min decreased from 95% to 5% B and maintained to 14.0 min. The injection volume was 5 μL.

While under lipidic profiling (hydrophobic compounds), chromatographic separation was performed on the Thermo C30 column (2.6μm, 2.1mm×100mm) with the mobile phases A of 0.04% acetic acid and 5 mM ammonium formate in acetonitrile/water (60:40, *v*:*v*) and mobile phase B of 0.04% acetic acid and 5mM ammonium formate in acetonitrile/isopropanol (10:90, *v:v*) at 45 ℃ and a flow rate of 0.35 mL/min. Gradient program was as follows: 0-3min increased from 20% to 50% B, 3-5min increased from 50% to 65% B, 5-9 min increased from 65% to 75% B, 9-15.5 min increased from 75% to 90% B. The injection volume was 2 μL.

**1.3 ESI-QTRAP-MS/MS Conditions**

After separation by UPLC, mass spectrometry was performed using a triple quadrupole-linear ion trap (QTRAP), equipped with an electrospray ionization ESI Turbo Ion-Spray interface (AB Sciex). LIT and triple quadrupole (QQQ) scans were acquired on a triple quadrupole-linear ion trap mass spectrometer (QTRAP). Except Applied Biosystems 4500 QTRAP was utilized for hydrophilic compounds profiling in discovery set, all the measurements were conducted by QTRAP® 6500+ LC-MS/MS System, equipped with an ESI Turbo Ion-Spray interface, operating in positive and negative ion mode and controlled by Analyst 1.6.3 software (AB Sciex).

The ESI source operation parameters were as follows: ion source, turbo spray; source temperature 550 °C; ion spray voltage (IS) 5.5kV under positive ion mode (or -4.5kV in negative ion mode); ion source gas I (GSI), gas II (GSII), curtain gas (CUR) were respectively set at 55, 60, and 25 psi; the collision gas (CAD) was medium. Instrument tuning and mass calibration were performed with 10 and 100 μmol/L polypropylene glycol solutions in QQQ and LIT modes, respectively. QQQ scans were acquired as MRM experiments with collision gas (nitrogen) set to 5 psi. Declustering potential (DP) and collision energy (CE) for individual MRM transitions was done with further DP and CE optimization. A specific set of MRM transitions were monitored for each period according to the compounds eluted within this period.

**1.4 Plasma Metabolites and lipids qualitative and quantitative analysis**

Qualitative analysis of the precursor ion and fragments spectra detected was carried out on the basis of [self-built](javascript:;) MWDB (metware database) with retention time and ion pairs, as well as the public database of metabolites information. We used MS/MS spectra to search against public databases to improve confidence in metabolite identification. Some of these substances are qualitatively analyzed with removing isotopic signals, repetitive signals containing K^+^ ions, Na^+^ ions, and NH4^+^ ions, as well as repeated signals of fragmented ions that themselves are of larger molecular weight. Metabolite and lipid structure resolution is referenced in existing mass spectrometry public databases such as MassBank (http://www.massbank.jp/)(1), HMDB (http://www.hmdb.ca/)(2), METLIN (http://metlin.scripps.edu/index.php)(3) and the Lipid maps Structure Database (LMSD, https://www.lipidmaps.org/data/structure/)(4). The metabolite identification was conducted by alignment to the reference standards in [self-built](javascript:;) database and public databases, and more information of the ionization modes and ion pairs of the metabolites and lipid species are provided in our previously published study (5). MetaboAnalyst (https://www.metaboanalyst.ca) (version 4.0)(6) and Kyoto Encyclopedia of Genes and Genomes (KEGG) database (http://www.genome.jp/kegg/) (7) was used to analyze the pathway enrichment for the identification of highly enriched metabolic pathways based on the significant metabolic markers.

Quantitation of metabolites was accomplished using multiple reaction monitoring (MRM) of triple quadrupole mass spectrometry. Under the MRM mode, the quadrupole rod first screened precursor ions (parent ions) of the target substance to exclude ions corresponding to other molecular weight substances to preliminarily eliminate the interference. The precursor ions were induced to ionize in the collision cell to form many fragment ions, fragment ions. The fragment ions are then filtered through the triple four-stage bar to select a characteristic fragment ion needed, which eliminates the interference of non-target ions, making the quantification of better accuracy and repeatability. The mass spectrum data were processed by Analyst 1.6.3 software (AB Sciex). After obtaining the metabolite spectrum analysis data of different samples, the peak area integral was performed for the mass spectrum peaks, and the integral correction was performed for the mass spectrum peaks of the same metabolite in different samples.

**1.5 Quality control**

Quality control (QC) samples were prepared with pooling aliquots of each of the individual plasma samples, which were extracted as described above. During instrumental running, one QC sample was inserted into every 10 samples to monitor the analysis reproducibility. Extracts of this pooled plasma sample were injected to access process variability. As an additional QC, water aliquots were extracted as part of the sample set to serve as process blanks for artifact identification. By overlapping display analysis of the total ion chromatogram (TIC) of different QC samples for mass spectrometric detection and analysis, the repeatability of the extraction and detection of metabolites can be judged, that is, technical repeatability. The high stability of the instrument provides an important guarantee for the repeatability and reliability of the data (**Figure S1**).

**1.6 Data Preprocessing**

For metabolomic and lipidomic analyses, raw signals with more than half of the missing rate in the QC samples (those with zero ion intensity) were removed. Missing metabolomic data were imputed by replacing the missing value with a minimum value of the metabolite quantified. To adjust signal drift, we applied the Quality Control–based Robust LOESS (LOcally Estimated Scatterplot Smoothing) Signal Correction (QC–RLSC) algorithm for analytical batch effect correction (8), which is an effective way to normalize the metabolic features to the QC samples within an analytical block. The dataset was then scaled by pareto scaling with procedures of mean centering and scaling to the square root of standard deviation (9). Then, the matrix was exported for further analysis.

# Supplementary Figures


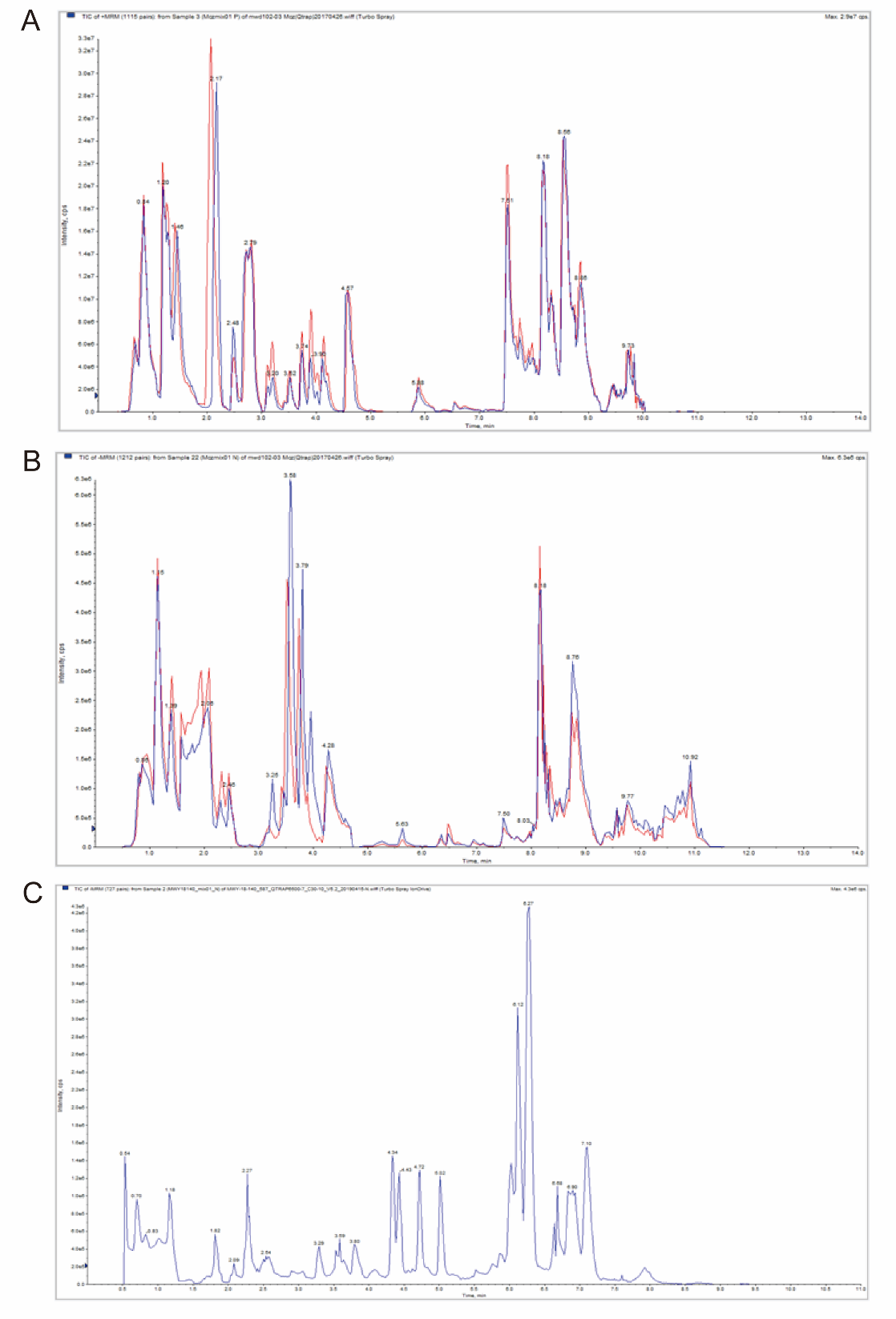


**Figure S1.** Representative total ion flow diagrams between different QC samples. **A** and **B** showed metabolic profiling under positive ion mode and negative ion mode, respectively; **C** presented lipid profiling under positive mode.


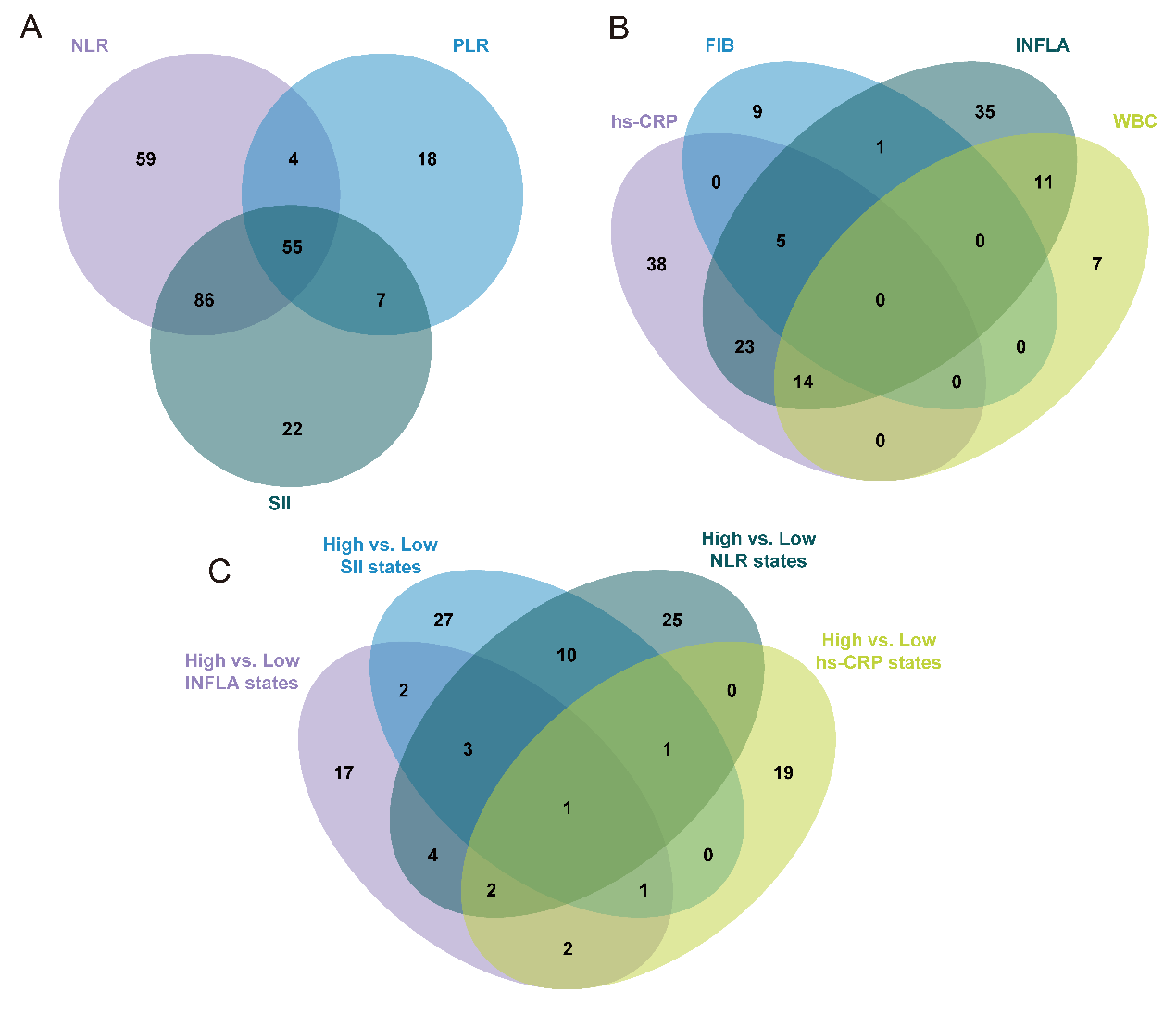


**Figure S2.** Venn diagrams presented the overlap in significantly associated metabolites, for each inflammatory marker in plasma of the SII **(A)** and INFLA **(B)** cohort, and for different inflammatory states defined with different markers **(C)**.

**References**

1. Horai H, Arita M, Kanaya S, Nihei Y, Ikeda T, Suwa K, et al. MassBank: a public repository for sharing mass spectral data for life sciences. *J Mass Spectrom* (2010) 45: 703-14. doi:10.1002/jms.1777

2. Wishart D S, Feunang Y D, Marcu A, Guo A C, Liang K, Vazquez-Fresno R, et al. HMDB 4.0: the human metabolome database for 2018. *Nucleic Acids Res* (2018) 46: D608-D617. doi:10.1093/nar/gkx1089

3. Smith C A, O'Maille G, Want E J, Qin C, Trauger S A, Brandon T R, et al. METLIN: a metabolite mass spectral database. *Ther Drug Monit* (2005) 27: 747-51. doi:10.1097/01.ftd.0000179845.53213.39

4. Sud M, Fahy E, Cotter D, Brown A, Dennis E A, Glass C K, et al. LMSD: LIPID MAPS structure database. *Nucleic Acids Res* (2007) 35: D527-32. doi:10.1093/nar/gkl838

5. Chen H, Wang Z, Qin M, Zhang B, Lin L, Ma Q, et al. Comprehensive Metabolomics Identified the Prominent Role of Glycerophospholipid Metabolism in Coronary Artery Disease Progression. *Front Mol Biosci* (2021) 8: 632950. doi:10.3389/fmolb.2021.632950

6. Chong J, Soufan O, Li C, Caraus I, Li S, Bourque G, et al. MetaboAnalyst 4.0: towards more transparent and integrative metabolomics analysis. *Nucleic Acids Research* (2018) 46: W486-W494. doi:10.1093/nar/gky310

7. Kanehisa M and Goto S. KEGG: kyoto encyclopedia of genes and genomes. *Nucleic Acids Res* (2000) 28: 27-30. doi:10.1093/nar/28.1.27

8. Luan H, Ji F, Chen Y and Cai Z. statTarget: A streamlined tool for signal drift correction and interpretations of quantitative mass spectrometry-based omics data. *Anal Chim Acta* (2018) 1036: 66-72. doi:10.1016/j.aca.2018.08.002

9. van den Berg R A, Hoefsloot H C, Westerhuis J A, Smilde A K and van der Werf M J. Centering, scaling, and transformations: improving the biological information content of metabolomics data. *BMC Genomics* (2006) 7: 142. doi:10.1186/1471-2164-7-142
